# Blood IgE and Eosinophils are not Reliable Predictors of Nasal Tissue Eosinophils in Chronic Rhinosinusitis with Nasal Polyposis

**DOI:** 10.1055/s-0044-1801316

**Published:** 2025-04-15

**Authors:** Judy Fan, William Daniels, Athenea Pascual, Changwan Ryu, Masih Sarafan, Miguel Soares Tepedino, Richard Louis Voegels, Marco Aurélio Fornazieri, Don Sin, Rogério Pezato, Andrew Thamboo

**Affiliations:** 1 Division of Otolaryngology, Head and Neck Surgery, Department of Surgery, Faculty of Medicine, University of British Columbia, Vancouver, BC, Canada; 2 Ear, Nose and Throat Research Lab, Department of Otorhinolaryngology and Head and Neck Surgery, Universidade Federal de São Paulo, São Paulo, SP, Brazil; 3 Department of Otolaryngology, Policlinica de Botafogo, Rhinology and Skull Base Division, Universidade do Estado do Rio de Janeiro, Rio de Janeiro, RJ, Brazil; 4 Department of Ophthalmology and Otorhinolaryngology, Universidade de São Paulo, São Paulo, SP, Brazil; 5 Department of Clinical Surgery, Universidade Estadual de Londrina, Londrina, PR, Brazil; 6 Division of Respiratory Medicine, Department of Medicine, Faculty of Medicine, University of British Columbia, Vancouver, BC, Canada

**Keywords:** rhinosinusitis, nasal polyps, inflammation, immunoglobulin E, eosinophils

## Abstract

**Introduction** Chronic rhinosinusitis with nasal polyposis (CRSwNP) is a chronic inflammatory condition of the paranasal sinuses that is mainly associated with type-2 inflammation. Immunoglobulin E (IgE) and eosinophils in blood and nasal tissue have been suggested as biomarkers for the prognosis and severity of CRSwNP as well as indications for biological treatment.

**Objective** The present study aims to assess the relationships between blood IgE concentration, blood eosinophil count, and nasal polyp eosinophil count in CRSwNP patients.

**Methods** The present study is retrospective. Nasal polyps from CRSwNP patients (n = 73) were fixed and embedded in paraffin for hematoxylin and eosin stain. Blood was collected to measure IgE concentration and eosinophil count.

**Results** Weak correlations were found between blood and tissue eosinophil counts ( *p* = 0.004, r = 0.367) as well as blood IgE concentration and blood eosinophil count ( *p* = 0.007, r = 0.372). There was no statistically significant correlation between blood IgE concentration and tissue eosinophil count. When dividing patients based on nasal polyp eosinophil count, blood eosinophil level was higher in the severely eosinophilic group than in the mildly eosinophilic group ( *p* = 0.002).

**Conclusion** Blood IgE and eosinophils are not reliable biomarkers to predict the inflammatory condition in CRSwNP. Further research is needed on the clinical roles of these biomarkers.

## Introduction

Chronic rhinosinusitis with nasal polyposis (CRSwNP) is a chronic inflammatory condition of the paranasal sinuses. Although its etiology is not fully understood, recent evidence has highlighted the importance of type-2 inflammation in the pathogenesis of CRSwNP. 1 2 Type-2 inflammation is clinically significant due to its close association with chronic inflammatory conditions (such as asthma and atopic dermatitis) and higher rates of revision surgery. 3 4 Hence, there has been a growing interest in monoclonal antibody biologics targeting the type 2-inflammatory cascade, including mepolizumab (anti-IL-5), omalizumab (anti-IgE), and dupilumab (anti-IL-4Rα). These biologics, approved by Health Canada and the FDA, demonstrated their efficacy in improving the clinical, endoscopic, and radiological symptoms of CRSwNP. 5 6 7 They provide an alternative for patients with comorbid allergic and respiratory conditions and/or did not respond to surgeries.

With the advent of biologics, the identification of biomarkers is needed to pinpoint specific CRSwNP endotypes that better respond to particular drugs. To address this need, eosinophil and IgE have been suggested as clinical biomarkers to predict the severity of typ-2 CRSwNP. 8 According to the European Position Paper on Rhinosinusitis and Nasal Polyps (EPOS) 2020, meeting one of the cut-off points, ≥ 10 tissue eosinophil/high-power field (eos/HPF), ≥ 250 u/l blood eosinophil, or ≥ 100 IU/ml total IgE, was considered evidence of type-2 inflammation and an indication for biological treatment in CRSwNP. 9 However, there have been conflicting findings in the literature about whether the eosinophil level in nasal tissue correlates with the eosinophil and IgE levels in blood, ranging from weak and 10 moderate 11 to strong 12 13 correlations. Furthermore, many studies first categorized CRSwNP patients into eosinophilic and mildly eosinophilic based on tissue eosinophil count, and then compared the blood biomarkers between the two groups. This could be questionable as the threshold for tissue eosinophilia is inconsistent in the literature that would range from > 5 eos/HPF 14 to ≥ 70 eos/HPF. 15 It is also hard to distinguish the clinical characteristics of patients with, for example, 10 eos/HPF and 99 eos/HPF as they are in the same group. Therefore, further research is required to determine whether peripheral eosinophil and serum IgE are reliable biomarkers for biological treatment. Our study aims to better understand if the eosinophil count in the nasal polyps of CRSwNP patients correlates with the eosinophil and IgE levels in blood by not grouping these patients into eosinophilic and mildly eosinophilic.

## Methods

The present study was developed by the Division of Otolaryngology—Head and Neck Surgery of two universities. The nasal polyps of CRSwNP patients (n = 73) with no history of sinus surgery were obtained during functional endoscopic sinus surgery performed at local hospitals. The study was approved by local research ethics boards. Patient consent was obtained before each sample collection. The diagnosis of CRSwNP was based on medical history, clinical examination, nasal endoscopy, and computed tomography (CT) of the paranasal sinuses according to EPOS 2020. 9 Patient blood was collected during surgery to quantify IgE concentration and eosinophil count based on laboratory guidelines from local hospitals.

### Nasal Polyp Histology

There was a 30-day washout period of systemic or topical steroidal antiinflammatory drugs prior to surgery. Nasal polyps from CRSwNP patients were obtained by forceps excision during surgery. They were weighed, fixed with 10% acetaldehyde, and maintained for 24 hours at room temperature. Previously fixed polyp tissues were embedded in paraffin and sliced into 4 μm sections with a microtome. All sections were affixed onto Superfrost Plus glass slides (Menzel Glaser, Braunschweig, Germany) and dried at 60° C for a few hours. For deparaffinization, the slides were washed successively in xylene 3 times for 10 minutes, 100% ethanol 2 times for 5 minutes, 90% ethanol 2 times for 5 minutes, and 70% ethanol 2 times for 5 minutes. The nuclei were stained with alum hematoxylin (Lillie-Mayer solution) (Eng Scientific, New Brunswick, New Jersey) for 5 minutes and rinsed with tap water. The sections were differentiated with 0.3% acid alcohol, rinsed with tap water and subsequently Scott tap water substitute (sodium hydrogen carbonate 10 g, magnesium sulfate 100 g, distilled water 5 L). The sections were stained with eosin solution (1% eosin Y 400 mL, 1% aqueous phloxine 40 mL, 95% alcohol 3,100 mL, and glacial acetic acid 16 mL) for 2 minutes, dehydrated and cleared. Histological examination was performed with a Leica DM2000 (Leica Camera AG, Wetzlar, Germany) binocular microscope at 400x magnification. The number of eosinophils per HPF was counted in an average of 10 fields of view selected from the most inflamed areas of nasal tissues. 16

### Statistical Analysis

Spearman's test, the Kolmogorov-Smirnov test, and the Kruskal-Wallis test were conducted to assess correlations, normal distributions, and between-group differences, respectively. A *p* -value ≤ 0.05 was considered statistically significant. All analyses were performed on IBM SPSS Statistics for Windows, version 28.0 (IBM Corp., Armonk, NY, USA).

## Results

### The Correlations Between Blood and Tissue Biomarkers

**Fig. 1:**
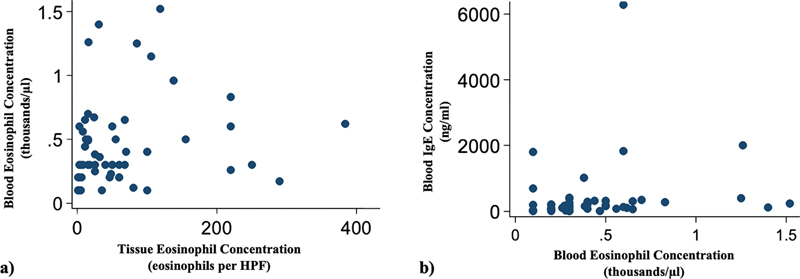
The correlations of biomarkers for chronic rhinosinusitis with nasal polyposis. ( **A** ) The correlation between eosinophil count in blood (thousands/µl) and eosinophil count (eosinophils per high power field) in nasal polyps ( *p* = 0.004, r = 0.367). ( **B** ) The correlation between eosinophil count in blood (thousands/µl) and IgE concentration (ng/ml) in blood ( *p* = 0.007, r = 0.372).

The eosinophil count in nasal polyps was found to poorly correlate with the eosinophil count ( *p* = 0.004, r = 0.367) in blood ( Fig. 1A ). No statistically significant correlation was found between the eosinophil count in nasal polyps and the IgE concentration in blood. A weak and positive correlation ( *p* = 0.007, r = 0.372) was found between eosinophil count and IgE concentration in blood ( Fig. 1B ).

### Group Comparisons Based on Tissue Eosinophil Count

**Fig. 2:**
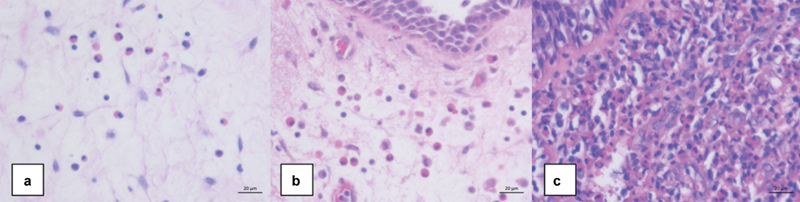
Hematoxylin-eosin-stained sections of nasal polyps from patients with chronic rhinosinusitis with nasal polyps demonstrating a) mild, b) moderate, and c) severe presence of eosinophils.

**Fig. 3:**
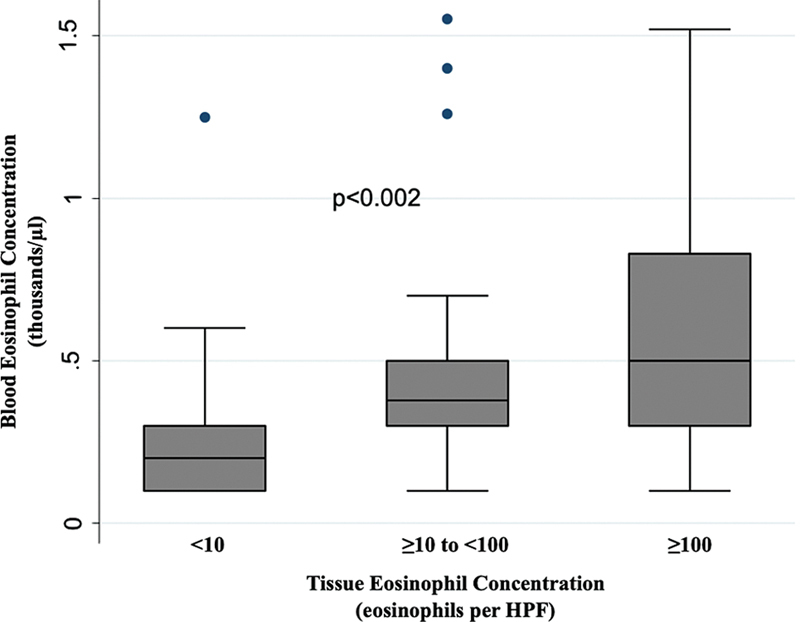
The comparisons of blood eosinophil count in three groups of patients with chronic rhinosinusitis with nasal polyps ( *p* < 0.002) based on the eosinophil count in nasal polyps: mildly eosinophilic < 10 eosinophils/high power field (eos/HPF), eosinophilic ≥ 10 eos/HPF and severely eosinophilic ≥ 100 eos/HPF.

The CRSwNP patients were categorized into three groups based on the eosinophil count in nasal polyps: mildly eosinophilic (< 10 eos/HPF), eosinophilic (≥ 10 eos/HPF), and severely eosinophilic (≥ 100 eos/HPF). Hematoxylin and eosin-stained sections of nasal polyps were illustrated in Fig. 2 . Blood eosinophil count in eosinophilic and severely eosinophilic CRSwNP patients were found to be higher ( *p* < 0.002) than the mildly eosinophilic group ( Fig. 3 ).

## Discussion

In our study, the eosinophil count in nasal polyps was found to poorly correlate (r = 0.367) with that in blood. No statistically significant correlation was detected between nasal polyp eosinophil count and blood IgE concentration. These findings are consistent with a retrospective study on 70 CRSwNP patients, where no statistically significant relationship between tissue and serum eosinophil counts was detected by Pearson correlation and group comparison (≤ 10 and >10 eos/HPF in nasal tissue). 10 Serum eosinophil level was also found to not correlate with the severity of CRSwNP based on olfactory symptoms, Lund-Mackay CT scores, asthma conditions and aspirin sensitivity. In another study, 92.4% of the 70 CRSwNP patients were found to have mild-to-severe blood eosinophilia, while only 36% showed mild tissue eosinophilia. 17 This finding demonstrated that the patient cohorts of high blood and tissue eosinophil counts do not necessarily overlap with each other, suggesting that serum eosinophil is not a predictor for tissue eosinophilia. Similar studies were also conducted on asthma patients, according to which blood IgE and eosinophil levels were found to be not reliable to predict airway inflammation, based on eosinophil counts in sputum, bronchoalveolar lavage, and endobronchial biopsy. 18 19 Moreover, our study showed that the eosinophil count poorly correlates with the IgE concentration in blood, which also suggested that blood may not be reflective of the inflamed environment in nasal polyps and could be impacted by other physiological activities.

Although numerous studies, including ours, are not in favor of adopting blood IgE and eosinophil as CRSwNP biomarkers, this topic is still under debate, and there are many other studies that detected a positive correlation between blood and tissue biomarkers. Yet, we noticed that among those studies, many categorized CRSwNP patients as eosinophilic and mildly eosinophilic. Patients with high mucosal eosinophil count (> 10 eos/HPF) were found to have higher peripheral eosinophil count, Lund-Mackay CT scores, and Lund-Kennedy endoscopic scores than patients with low mucosal eosinophil count (≤ 10 eos/HPF). 12 20 21 We proposed that the positive association detected in those studies was attributed by the grouping of CRSwNP patients based on their tissue eosinophil count. When we divided our patients into 3 groups: mildly eosinophilic (< 10/HPF), eosinophilic (≥ 10 and < 100/HPF), and severely eosinophilic (≥ 100/HPF), we also found a statistically significant relationship ( *p* < 0.002) between blood and tissue eosinophil levels. We believe this approach may not be accurate. For example, the clinical characteristics of patients with 10 eos/PHF and 99 eos/PHF are hardly differentiable, as they are in the same group. Meanwhile, patients with 9 eos/PHF and 10 eos/PHF might be associated with distinct endotypes as they are in separate groups, despite the difference being merely 1 eos/PHF. Furthermore, the threshold for tissue eosinophilia varies across the literature, ranging from > 5 eos/PHF 14 to ≥ 70 eos/HPF. 15 These factors and uncertainties to the relationship between tissue and blood biomarkers; thus, we suggest treating tissue eosinophil count as a continuous variable to better represent this relationship. We recognize that the sample size of this study is small (n = 73); a larger sample may be necessary to enhance generalizability and robustness of the conclusion. However, our study still contributes to the literature as one of the very few studies that do not group patients based on nasal polyp eosinophil count. Specifically in our study, we used Spearman correlation ( Fig. 1 ) and found that nasal polyp eosinophil count does not correlate with serum IgE level, and poorly correlates with peripheral eosinophil count. Further studies with large cohorts will be needed to validate and extend the implications of current findings.

Our study raised concerns about relying solely on blood eosinophil and IgE levels as evidence of type-2 inflammation and indications for biological treatment. Currently, in Canada and the US, no consensus has been established on the clinical role of systematic biomarkers in CRSwNP patients. One limitation of our study is that we did not further explore the correlation between these markers and disease severity, as assessed by clinical findings such as the Lund-Mackay computed tomography (CT) scores. Further research should focus on understanding this relationship for accurately assessing the prognosis and severity of CRSwNP, as well as responses to biologics. These studies will provide important guidance for targeted therapy, clinical practice, and disease management of CRSwNP and other type-2 inflammatory diseases including asthma and atopic dermatitis.

## Conclusion

Our study suggests that blood IgE and eosinophils are not reliable biomarkers to predict the inflammatory condition in CRSwNP. Further investigations are needed into the clinical roles of these biomarkers in biological treatment.
